# Supercritical Extraction and Identification of Bioactive Compounds in *Dryopteris fragrans* (L.) Schott

**DOI:** 10.3390/ph18030299

**Published:** 2025-02-21

**Authors:** Mayya P. Razgonova, Zhanna M. Okhlopkova, Muhammad A. Nawaz, Polina S. Egorova, Kirill S. Golokhvast

**Affiliations:** 1 N.I. Vavilov All-Russian Institute of Plant Genetic Resources, B., Saint-Petersburg 190000, Russia; golokhvast@sfsca.ru; 2 Far Eastern Federal University, Vladivostok 690950, Russia; 3 Department of Biology, North-Eastern Federal University, Yakutsk 677000, Russia; zhm.okhlopkova@s-vfu.ru; 4 Advanced Engineering School «Agrobiotek», National Research Tomsk State University, Tomsk 634050, Russia; 5 Yakutsk Botanical Garden, Institute for Biological Problems of Cryolithozone, Siberian Branch, Russian Academy Sciences, Yakutsk 677007, Russia; egorovaps@ibpc.ysn.ru; 6 Siberian Federal Scientific Centre of Agrobiotechnology, Russian Academy of Sciences, Presidium, Krasnoobsk 633501, Russia

**Keywords:** *Dryopteris fragrans* (L.), tandem mass spectrometry, supercritical CO_2_ extraction, polyphenols, metabolome

## Abstract

**Background:** This is a comparative metabolomic study of the medicinal plant *Dryopteris fragrans* (L.) Schott from the family *Dryopteridaceae* Herter (or *Aspidiaceae* Mett. ex Frank) growing under cold pole conditions in the Oymyakon region of the Republic of Sakha (Yakutia). **Methods:** The aerial parts of *D. fragrans* were subjected to extraction using supercritical CO_2_ extraction and maceration methods. Several experimental conditions were investigated, including a pressure range of 50–300 bar and a temperature range of 31–60 °C. A 1% volume of ethanol was used as a co-solvent in the liquid phase of the extraction. **Results:** The most effective *D. fragrans* extraction conditions were 200 Bar pressure and a temperature of 55 °C. Tandem mass spectrometry was used to detect the target analytes. A total of 141 bioactive compounds (86 compounds from the polyphenol group and 55 compounds from other chemical groups) were tentatively identified in extracts of aerial parts of *D. fragrans*. Among these, thirty chemical constituents from the polyphenol group were identified for the first time. Other compound classes that were newly identified in *D. fragrans* include naphthoquinones (5,8-dihydroxy-6-methyl-2,3-dihydro-1,4-naphthoquinone, 1,8-dihydroxy-anthraquinone, 1,4,8-trihydroxyanthraquinone, chrysophanol, etc.), diterpenoids (tanshinone IIa, cryptotanshinone, isocryptotanshinone II, tanshinone IIb, etc.), polysaccharides, triterpenoids, and sesquiterpenes. **Conclusions:** These results highlight that *D. fragrans* is rich in bioactive compounds and put forward several newly detected compounds for further investigation.

## 1. Introduction

**Figure 1:**
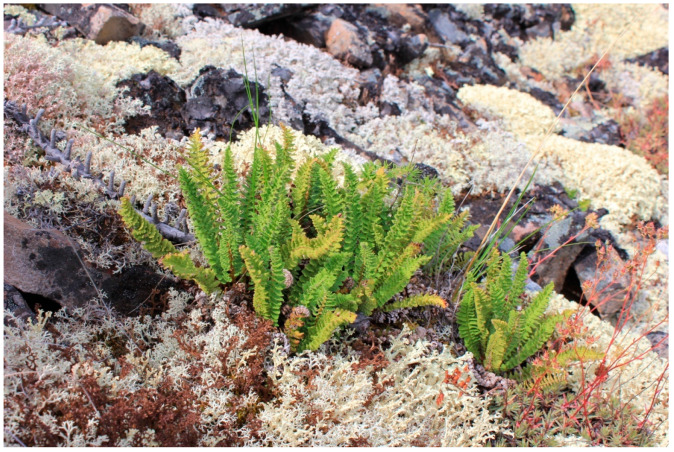
*Dryopteris fragrans* (L.) Schott. (Oymyakon Highlands; photo by Okhlopkova Zh. M.; July 2019).

*Dryopteris fragrans* (L.) Schott is a perennial, grassy, low-growing fern with a short rhizome belonging to the Dryopteridaceae family (or Aspidiaceae Mett. ex Frank) (Figure 1). *D. fragrans* is distributed across the Far East and Eastern Siberia; outside of Siberia, it is found in Northern Europe, Northeast Asia, and North America (Figure 1) [1].

In Yakutia, it is found in all floristic regions along stony larch woodlands, thickets of Siberian dwarf pines, and stone runs [2,3]. Infusions and tinctures of *D. fragrans* (L.) Schott were tested by Lebedev V.V. in laboratory animals and calves under production conditions. The infusion of both animals stimulated bile secretion slightly, whereas tinctures had a general anti-inflammatory effect and a pronounced vasoconstrictor effect. Up to 97.4% of calves with dyspepsia were cured via infusion and tincture of fragrant woodfern [4,5]. *D. fragrans* is used as a traditional medicine for the treatment of diseases such as psoriasis, rashes, dermatitis, Barbiers, and arthritis. The root of the fragrant fern is used by the local Yakut people as an anthelmintic agent, while the leaves of *D. fragrans* are administered to children with “fever in the abdomen” [2,4,5,6]. Previous research revealed that its major secondary metabolites, such as photoglycines, terpenes, lignans, phenolic glycosides, and essential oils, might be responsible for the pharmacological effects observed in clinical settings [7,8,9,10,11]. In traditional Yakutian medicine, a decoction or infusion of the leaves of the fragrant fern is used to treat gastric diseases, paralysis, coughs, and bone pain [4,6]. One popular name for this medicinal plant is “The herb that the wolf eats”.

Many researchers have studied the constituent compounds of *D. fragrans* and their potential applications.

Using spectroscopic analysis, Ito H. et al. (1997) elucidated the structure of a new sesquiterpenoid, dryofragin, isolated from *D. fragrans* [7]. Similarly, Kuang H. et al. (2008) isolated a new phenolic glycoside, 3,3,5-dimethyl-6-hydroxy-2-methoxy-4-*O*---*D*-glucopyranosyl-oxy-acetophenone, from the aerial parts of *D. fragrans* and used spectroscopic methods to identify its structure [8]. New sesquiterpene glucosides were also isolated from an aqueous extract of the aerial parts of *D. fragrans*, identified as 3β, 11-dihydroxy-drim-8(12)-en-11-*O*-β-*D*-glucopyranoside, 3β, 11-dihydroxy-drim-8(12)-en-3-*O*-β-*D*-glucopyranoside, and 11,14-dihydroxy-drim-8(12)-en-11-*O*-β-*D*-glucopyranoside using high-performance liquid chromatography (HPLC), high-resolution electrospray ionization mass spectrometry (HRESIMS), and 1D and 2D nuclear magnetic resonance (NMR) analysis [9].

One new coumarin, dryofracoumarin A, 8-hydroxyl-4-isopropyl-7-methyl-6-methyl-2H-benzopyran-2-one, and six known compounds, esculetin, isoscopoletin, methylphlorbutyrophenone, aspidinol, albicanol, and (E)-4-(3,4-dimethoxyphenyl)but-3-en-1-ol, were isolated from *D. fragrans*. The new coumarin, as well as esculetin and isoscopoletin, showed significant cytotoxic activity against two cell lines (A549 and MCF7). Scientists have suggested that these active compounds may be promising cancer treatments [10]. Similarly, one new sesquiterpene, 3-*O*-β-*D*-glucopyranosylalbicanol-11-*O*-β-*D*-glucopyranoside, and six known compounds—Dihydroconiferylalcohol, (E)-3-(4-hydroxyphenyl) acrylic acid, esculetin, and 5,7-dihydroxy-2-hydroxymethylchromone—isolated from *D. fragrans* showed activity against *Microsporum canis* and *Epidermophyton floccosum* [11]. Furthermore, a detailed review highlighted the phytochemical composition and biological activity of plants of the genus *Dryopteris* [12]. Two new phenolic glycosides—frachromone C and dryofracoulin A—and one known compound, undulatoside A, were isolated from an aqueous extract of *D. fragrans* collected in Wudalianchi, Heilongjiang Province, China. The structures of these compounds were elucidated using a combination of 1D and 2D NMR, HRMS, and chemical analysis [13].

The cytotoxic constituents of *D. fragrans* have also been reported. For example, the methanol extract of the aerial parts of *D. fragrans* was used to isolate dryofragone, dryofracoumarin B, and other known compounds. These compounds exhibited modest cytotoxicity toward the human HeLa cell line, with an IC50 value below 30 µM, by using the 3-(4,5-dimethyl-2-thiazolyl)-2,5-diphenyl-2H-tetrazolium bromide assay [14]. Apart from traditional cytotoxicity studies, the use of in silico methods to understand the health beneficial activities of metabolites from this plant has also been reported. The antitumor activity of a phenolic compound isolated from *D. fragrans* was studied using molecular docking. Different variants of bicyclic phloroglucinol were considered in relation to A549, HepG2, and MCF-7; the ones with high binding affinity and good efficacy were selected as promising antitumor compounds [15]. Among other phenolic compounds, two new sesquiterpenoid glycosides, drioptesterpene A and drioptesterpene B, isolated from the aqueous extract of *D. fragrans*, exhibited anti-inflammatory activities [16]. Additionally, various modifications of the phloroglucinol compounds isolated from *D. fragrans* have been investigated. For example, various phloroglucinol derivatives have been synthesized with certain antiproliferative activities on cancer cell lines, as well as with the induction of apoptosis, in a concentration-dependent manner [17].

Currently, various methods are used to extract bioactive compounds from medicinal plants on a laboratory or commercial scale. A detailed review of the recent advances in the methods indicated that supercritical (SC) fluid extraction is one of the best techniques for the extraction of natural chemical constituents [18]. However, the use of such an efficient method has not been realized for *D. fragrans.* Most studies on *Dryopteris* species have reported the use of ethanol extraction [10,19,20]. Owing to its non-polar, gal-like, liquid-like properties and the ability to extract heat-sensitive compounds [18], we report its utility in *D. fragrans.*

**Figure 2:**
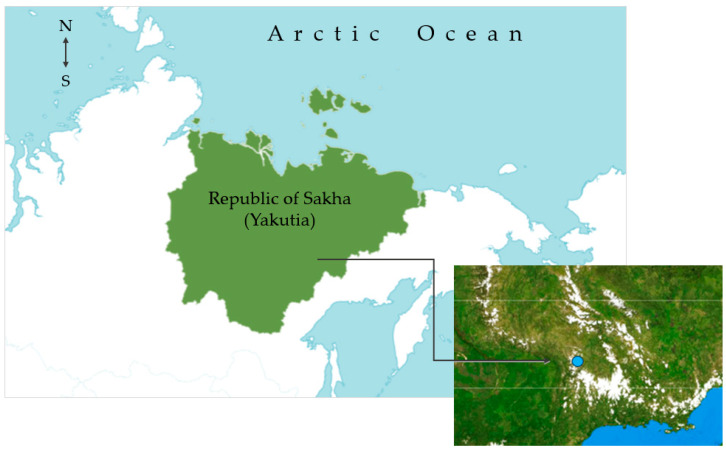
*D. fragrans* (L.) Schott specimen collection site, the area of the Kyubeme of the Oymyakon district of the Republic of Sakha (Yakutia) [blue circle in the satellite view represents the coordinates of the sampling site. N 63.417262°, E 140.610894°].

As the pharmaceutical industry is increasingly focusing on the search for new health beneficial compounds from a number of medicinal plants, it is necessary to determine the phytochemical composition of *D. fragrans* growing in the territory of Yakutia. Here, we conducted a detailed study of the biochemical composition of the aerial part of *D. fragrans* collected from the Kyubeme locality of the Oymyakon district of the Republic of Sakha (Yakutia) [N 63.417262°, E 140.610894°] during the expedition work in 2019–2020 (Figure 2).

## 2. Results

### Supercritical CO_2_ Extraction from the Aerial Parts of D. fragrans Plant

**Figure 3:**
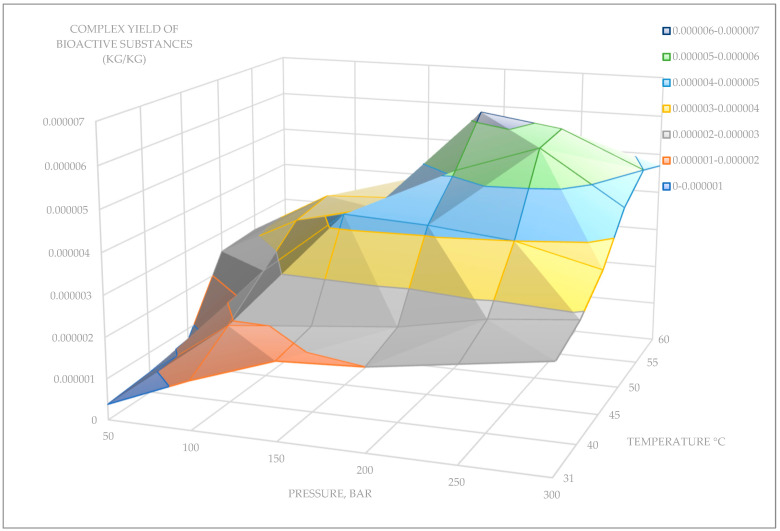
A 3D graph of the yield of bioactive substances *D. fragrans* under different supercritical extraction conditions.

The aerial parts of *D. fragrans* were examined via supercritical CO_2_ (SC-CO_2_) extraction under different extraction conditions. The SC pressures applied ranged from 50 to 250 bar, and the extraction temperature ranged from 31 to 70 °C. The co-solvent EtOH was used in an amount of 1% of the total solvent amount. Below is a 3D plot of the yield of bioactive substances *D. fragrans* under different SC extraction conditions (Figure 3).

Table 1 shows the yield of bioactive substances from aerial parts of *D. fragrans* via CO_2_ extraction.

The maximum yield of bioactive substances from aerial parts of *D. fragrans* was observed under the following extraction conditions:

− Pressure of 200 bar, extraction temperature of 55 °C, extraction time of 1 h. The yield of biologically active substances was 0.0000062 kg/0.0001 kg of plant sample; the proportion of the EtOH modifier was 2%.
− Pressure of 250 bar, extraction temperature of 55 °C, extraction time of 1 h. The yield of biologically active substances was 0.0000059 kg/0.0001 kg of plant sample; the proportion of the EtOH modifier was 2%.

## 3. Discussion

### 3.1. Global Metabolome Profile of D. fragrans

The structural identification of each compound was performed on the basis of its mass and MS/MS fragmentation via tandem mass spectrometry. The chemical constituents were identified by comparing their retention index, mass spectra, and mass spectrometry fragmentation with a home-library database built by the Group of Biotechnology, Bioengineering and Food Systems at the Far-Eastern Federal University (FEFU) based on data from other spectroscopic techniques, such as nuclear magnetic resonance, ultraviolet spectroscopy, and mass spectrometry, as well as data from the literature that are continuously updated and revised. According to the classification developed by Schymanski et al. [18], the FEFU database belongs to the second level, which includes two components: metabolite annotation level B(i): accurate matching of FEFU database based on MS/MS secondary spectrum library; B(ii): accurate matching of the MS/MS secondary spectrum library based on computer simulation. We were able to tentatively identify 141 compounds from the extracts of the aerial parts of *D. fragrans*, 86 compounds from the polyphenol group, and 55 compounds from other compound classes. All tentatively identified polyphenols and other compounds, along with molecular formulae and MS/MS data for *D. fragrans*, are summarized in Appendix A, Table A1. The polyphenols identified in our study were categorized as flavones, flavonols, anthocyanidins, phenolic acids, lignans, coumarins, stilbenes, etc. In total, the polyphenol metabolites identified in our study belonged to 19 compound classes. The highest number of metabolites were flavones (27), followed by flavonols (16), anthocyanins (13), flavan-3-ols (3), phenolic acids (13), stilbenes (2), coumarins (6), lignans (2), etc. These numbers indicate that *D. fragrans* extracts are rich in flavonoids. The highest number of chemical compounds from other groups are naphthoquinones (6), diterpenoids (5), and phloroglucinol derivatives (6).

**Figure 4:**
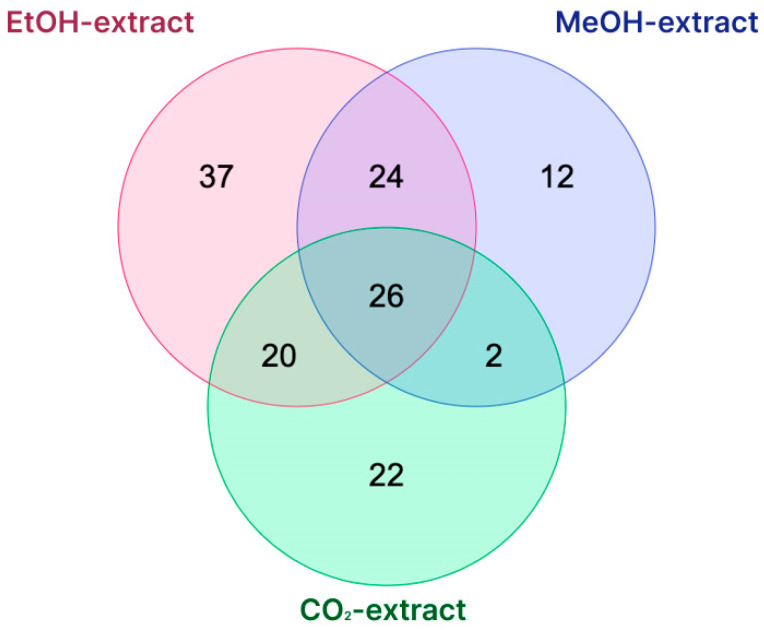
Venn diagram showing number of common and specific compounds in *D. fragrans*.

A Venn diagram showing the similarities and differences in the presence of various chemical groups in three extracts of *D. fragrans* (ethanol extract, methanol extract, and SC-CO_2_ extract) is shown in Figure 4. Twenty-six compounds were commonly identified from the three extracts of *D. fragrans*. These compounds belong to compound classes such as flavones, flavonols, anthocyanins, and phlorodlucinol derivatives, such as 1,3-dimethyluric acid, 8-dihydroxy-anthraquinone, albaspidin PB, flavaspidic acid BB, saroaspidin A, chlorogenic acid, chrysoeriol 6-*C*-glucoside, chrysoeriol 8-*C*-glucoside, chrysophanol, delphinidin 3-*O*-β-galactoside, delphinidin 3-*O*-hexoside, etc. The polyphenols, phlorodlucinol derivatives, naphthoquinones, and anthraquinones are major active compounds in extracts of *D. fragrans*. Overall, the applied methods were able to detect 107 (EtOH extract of *D. fragrans*), 64 (MeOH extract of *D. fragrans*), and 70 (CO_2_ extract of *D. fragrans*) compounds (Table 2).

In addition, we used the Jaccard index to represent the similarities and differences of bioactive substances in different extracts of *D. fragrans* (Table 3). The Jaccard index, also known as the Jaccard similarity coefficient, is a statistic used to evaluate the similarity and diversity of sets of samples [21,22].

Table 3 shows that the highest degree of similarity is present between the EtOH extract and MeOH extract—0.4132.

### 3.2. Flavones

**Figure 5:**
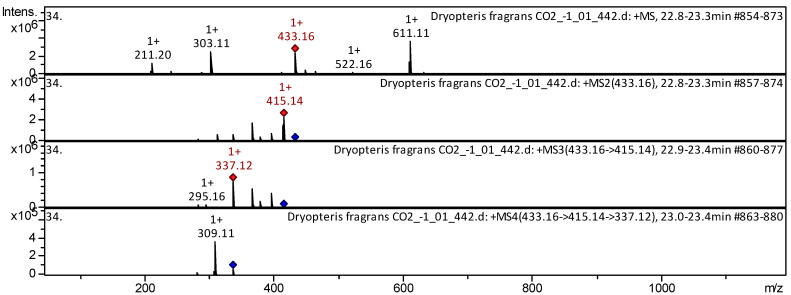
CID-spectrum of vitexin from aerial parts of *D. fragrans*, *m*/*z* 433.16. At the top is the MS scan in the range of 100–1700 *m*/*z*, and at the bottom are the fragmentation spectra (from top to bottom): MS^2^ of the protonated ion of vitexin (433.16 *m*/*z*, red diamond), MS3 of the fragment ion 433.16→415.14 *m*/*z*, and MS4 of the fragment ion 433.16→415.14→337.12 *m*/*z.*

The flavones dihydroxyflavone, formononetin, apigenin, trihydroxy(iso)flavone, luteolin, herbacetin, trimethoxy flavone, pentahydroxy dimethoxyflavone, vitexin, isovitexin, genistein 8-*C*-glucoside, etc. (compounds **1**–**27**; Appendix A Table A1) have already been characterized as a component of *Triticum aestivum* [23], *Dryopteris ramosa* [24], *Aspalathus linearis* [25], *Lonicera japonica* [26], *Passiflora incarnata* [27], and Mexican lupine species [28] (Appendix A, Table A1). The CID-spectrum (collision-induced spectrum) in positive ion modes of flavone vitexin from aerial parts of *D. fragrans* (CO_2_ extract) is shown in Figure 5.

The [M+H]+ ion produced a fragment ion: *m*/*z* 415.14 (Figure 5). The fragment ion with *m*/z 415.14 produced two characteristic daughter ions: *m*/*z* 337.12 and *m*/*z* 295.16. The *m*/z 337.12 daughter ion produced a characteristic *m*/*z* 309.12 ion. Detailed mass spectrometry of vitexin has been reported in studies of *D. ramosa* [24] and *T. aestivum* [23].

### 3.3. Flavonols

**Figure 6:**
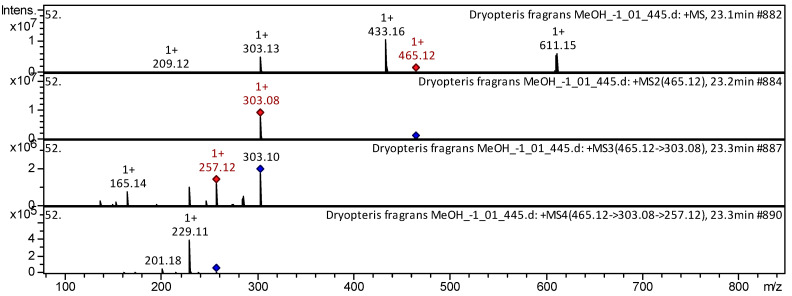
CID-spectrum of quercetin *3*-*O*-glucoside from aerial parts of *D. fragrans*, *m*/*z* 465.12. At the top is the MS scan in the range of 100–1700 *m*/*z*, and at the bottom are the fragmentation spectra (from top to bottom): MS^2^ of the protonated ion of quercetin 3-*O*-glucoside (465.12 *m*/*z*, red diamond), MS3 of the fragment ion 465.12→303.08 *m*/*z*, and MS4 of the fragment ion 465.12→303.08→257.12 *m*/*z.*

The flavonols quercetin, myricetin, kaempferol 3-*O*-pentoside, quercetin 3-*O*-pentoside, quercetin 3-D-xyloside, astragalin, hyperoside, myricitrin, rutin, etc. (compounds **28**–**43**; Appendix A Table A1) have already been characterized as a component of *Ribes meyeri* [29]; *Ribes magellanicum* [30]; *L. japonica* [26]. The CID-spectrum in positive ion modes of flavonol quercetin 3-*O*-glucoside from aerial parts of *D. fragrans* (MeOH extract) is shown in Figure 6.

The [M+H]+ ion produced one fragment ion: *m*/*z* 303.08 (Figure 6). The fragment ion with *m*/z 303.08 produced two characteristic daughter ions: *m*/*z* 257.12 and *m*/*z* 165.14. The daughter ion with *m*/z 257.12 produced two characteristic ions: *m*/*z* 229.11 and *m*/*z* 201.18. Detailed mass spectrometry of quercetin 3-*O*-glucoside has been reported in articles about *T. aestivum* [23], *R. meyeri* [29], *Lonicera japonicum* [26], *Cranberry* [31], Andean blueberry [32], and *Citrus sinensis* [33].

### 3.4. Anthocyanins

**Figure 7:**
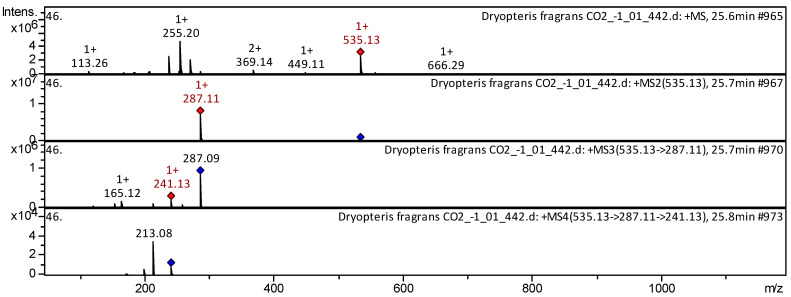
CID-spectrum of cyanidin 3-(6”-malonylglucoside) from aerial parts of *D. fragrans*, *m*/*z* 535.12. At the top is the MS scan in the range of 100−1700 *m*/*z;* at the bottom are the fragmentation spectra (from top to bottom): MS^2^ of the protonated ion of cyanidin *3-(6”*-malonylglucoside) (535.13 *m*/*z*, red diamond), MS3 of the fragment ion 535.13→287.11 *m*/*z*, and MS4 of the fragment ion 535.13→287.11→241.13 *m*/*z.*

Cyanidin-3-*O*-glucoside (compound **50**), cyanidin 3-*O*-hexoside (compound **51**), cyanidin 3-*O*-β-galactoside (compound **52**), delphinidin 3-*O*-glucoside (compound **53**), cyanidin 3-(6”-malonylglucoside) (compound **56**), etc. (compounds **49**–**60**, Appendix A Table A1), have already been characterized as a component of Andean blueberry [32], *Ribes dikuscha* [34], *Zostera marina* [35], *Medicago varia* [36], *T. aestivum* [37], and many other plant species whose organs (mainly fruits) accumulate pigments exhibiting a range of colors. The CID-spectrum in positive ion modes of cyanidin 3-(6”-malonylglucoside) from extracts from aerial parts of *D. fragrans* (CO_2_ extract) is shown in Figure 7.

The [M+H]^+^ ion produced one fragment ion: *m*/*z* 287.11. (Figure 7). The fragment ion with *m*/*z* 287.11 produced two characteristic daughter ions: *m*/*z* 241.13, and *m*/*z* 165.12. The anthocyanin cyanidin 3-(6”-malonylglucoside) glucoside has been tentatively identified in the extracts from several plant species, some of which are mentioned at the beginning of this paragraph along with others, such as *Z. marina* [35], *M. varia* [36], and *T. aestivum* [37].

### 3.5. Diterpenoids

**Figure 8:**
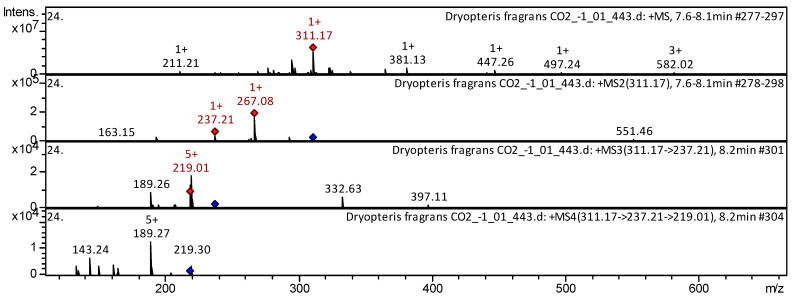
CID-spectrum of tanshinone IIB from aerial parts of *D. fragrans*, *m*/*z* 311.17. At the top is the MS scan in the range of 100−1700 *m*/*z;* at the bottom are the fragmentation spectra (from top to bottom): MS2 of the protonated ion of tanshinone IIB (311.17 *m*/*z*, red diamond), MS3 of the fragment ion 311.17→267.08 *m*/*z*, and MS4 of the fragment ion 311.17→267.08→219.01 *m*/*z.*

Tanshinone IIA (compound **109**), cryptotanshinone (compound **110**), isocryptotanshinone II (compound **111**), tanshinone IIB (compound **112**), and 7-dehydroabietic acid (compound **113**) (Appendix A Table A1) have already been characterized as a component of Huolisu Oral Liquid [38], Chinese herbal formula Jian-Pi-Yi-Shen pill [39], *Radix Salviae* [40]. The CID-spectrum in positive ion modes of tanshinone IIB from extracts from aerial parts of *D. fragrans* (CO_2_ extract) is shown in Figure 8.

The [M+H]^+^ ion produced three fragment ions: *m*/*z* 267.08, *m*/*z* 237.21, and *m*/*z* 163.15. (Figure 8). The fragment ion with *m*/*z* 267.08 produced two characteristic daughter ions: *m*/*z* 219.01 and *m*/*z* 189.26. Detailed mass spectrometry of tanshinone IIB has been reported in *Salviae Miltiorrhizae* [41] and Huolisu Oral Liquid [38].

### 3.6. Phlorodlucinol Derivatives

**Figure 9:**
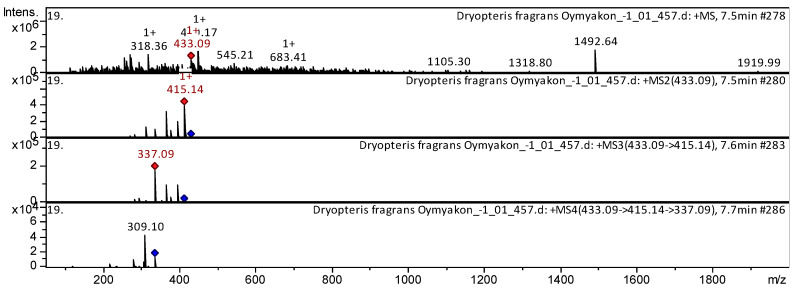
CID-spectrum of aspidin AB from aerial parts of *D. fragrans*, *m*/*z* 311.17. At the top is the MS scan in the range of 100−1700 *m*/*z;* at the bottom are the fragmentation spectra (from top to bottom): MS2 of the protonated ion of aspidin AB (311.17 *m*/*z*, red diamond), MS3 of the fragment ion 311.17→267.08 *m*/*z*, and MS4 of the fragment ion 311.17→267.08→219.01 *m*/*z.*

Disflavaspidic acid PB (compound **122**), aspidin AB (compound **123**), and albaspidin PP (compound **124**) (Appendix A Table A1) have already been characterized as a component of *D. fragrans* [20]. The CID-spectrum in positive ion modes of aspidin AB from extracts from aerial parts of *D. fragrans* (CO_2_ extract) is shown in Figure 9.

The [M+H]^+^ ion produced three fragment ions: *m*/*z* 267.08, *m*/*z* 237.21, and *m*/*z* 163.15. (Figure 9). The fragment ion with *m*/*z* 267.08 produced two characteristic daughter ions: *m*/*z* 219.01 and *m*/*z* 189.26. The detailed mass spectrometry of aspidin AB has been reported in an article about *D. fragrans* [20].

### 3.7. Newly Identified Chemical Compounds in D. Fragrans

Among the identified metabolites in the three *D. fragrans* extracts (EtOH extract, MeOH extract, and SC-CO_2_ extract), thirty compounds from the polyphenol group and 21 compounds from other chemical groups were identified for the first time. The newly identified polyphenols include flavones (dihydroxyflavone, formononetin, luteolin, herbacetin, genistein 8-*C*-glucoside, genistein 6-*C*-glucoside, acacetin 8-*C*-glucoside, luteolin C-hexoside, and eriodictyol 7-*O*-glucuronide), flavonols (astragalin, quercitrin, and myricitrin), anthocyanins (cyanidin 3-(6″-malonylglucoside, delphinidin-3-*O*-(6″-O-malonul)-β-D-glucoside, and delphinidin-3-*O*-(6-*O*-p-coumaroyl) glucoside), stilbenes pinosylvin and resveratrol, coumarins umbelliferone, fraxetin, 4/6,8-Dihydro-5,7-dihydroxy-2-*oxo*-2H-1-benzopyran-3-acetic acid, umbelliferone hexoside, tomenin, etc. Interestingly, the other compound classes that we also tentatively identified in *D. fragrans* are naphthoquinones (5,8-Dihydroxy-6-methyl-2,3-dihydro-1,4-naphthalenedione, 1,8-Dihydroxy-anthraquinone, 1,4,8-Trihydroxyanthraquinone, chrysophanol, etc.), diterpenoids (tanshinone IIA, cryptotanshinone, isocryptotanshinone II, tanshinone IIB, etc.), polysaccharides, triterpenoids, sesquiterpenes, etc. (Appendix A Table A1).

Biologically active compounds from the aerial parts and underground parts of plants are efficiently extracted using organic solvents, but the resulting extracts at the final stage require additional purification from traces of the solvents used. Supercritical CO_2_ extraction is declared as “green extraction” and is effectively used in various food processing processes as an alternative to traditional extraction methods [42,43]. With SC extraction, the resulting products do not contain residues of organic solvents, which are present in conventional extraction methods where solvents are often toxic or can be toxic. A low extraction temperature, easy removal of solvent from the final product, and high selectivity are the main attractive features of SC technology, leading to a significant increase in research opportunities for application in the food and pharmaceutical industries. Our research group has successfully applied the SC extraction method to extract bioactive components from *Z. marina* [35]. We also investigated the effects of SC-CO_2_ extraction parameters and the quality of *Panax ginseng* raw material on the yield of ginsenosides in the extraction of ginseng root [44]. Supercritical extraction has been successfully applied in the extraction of East Sikhotinsky rhododendron (*Rhododendron sichotense*) and East Siberian rhododendron (*Rhododendron adamsii*), which also showed that the SC-CO_2_ extraction is an effective method and provides additional opportunities for research [45]. Thus, the use of SC-CO_2_ extraction is an effective approach for the extraction of bioactive compounds. The results obtained in the extraction of *D. fragrans* are consistent with these reports that SC-CO_2_ is a useful approach for the extraction and study of bioactive compounds.

## 4. Materials and Methods

### 4.1. Materials

The aerial parts of *D. fragrans* plant were collected in the area of the Kyubeme, Oymyakon district, Republic of Sakha (Yakutia) [N 63.417262°, E 140.610894°] during the expedition work in 2019–2020. The plant material was dried on a horizontal surface in a ventilated room with periodic mixing. Before extraction, the dried material (the aerial parts) of *D. fragrans* was stored in a refrigerator at 4−6 °C. All samples complied with the morphological standards of the Pharmacopoeia of the Eurasian Economic Union [46].

### 4.2. Chemicals and Reagents

HPLC-grade acetonitrile was purchased from Fisher Scientific (Southborough, UK), and MS-grade formic acid was purchased from Sigma-Aldrich (Steinheim, Germany). Ultrapure water was prepared from a Siemens ultra clear (Siemens water technologies, Günzburg, Germany), and all other chemicals were analytical grade.

### 4.3. Extraction

Supercritical CO_2_ extraction was performed using the SFE-500 SC pressure extraction system (Thar SCF Waters Corporation, Milford, MA, USA). System options include co-solvent pump (Thar Waters P-50 High Pressure Pump, Waters Corporation, Milford, MA, USA) for extraction of polar samples, CO_2_ flow meter (Siemens AG, Günzburg, Germany) to measure the amount of CO_2_ supplied to the system, and multiple extraction vessels to extract different sample sizes or to increase the throughput of the system. The flow rate was 10−25 mL/min for liquid CO_2_ and 1.00 mL/min for EtOH. Extraction samples of 200 g aerial parts of *D. fragrans* were used. Extraction time was counted after reaching working pressure and equilibrium flow and was 60−90 min for each sample. This method of SC extraction of plant matrices was previously tested by our team on aerial and/or underground parts of different plant species [35,42,43].

### 4.4. Liquid Chromatography

We used high-performance liquid chromatography as well as mass spectrometry for the detection and identification of compounds in the extracts of *D. fragrans*, as reported in our earlier work [33,34].

High-performance liquid chromatography was performed using Shimadzu LC-20 Prominence HPLC (Shimadzu, Kyoto, Japan) equipped with a UV sensor and C18 silica reverse-phase column (4.6 × 150 mm, particle size: 2.7 μm). The mobile phase eluent A was deionized water containing 0.1% formic acid, and eluent B was acetonitrile containing 0.1% formic acid. Gradient elution was started at 0–2 min; 0% eluent B at 2−50 min, 0−100% B; and control washing at 50–60 min, 100% B. The mobile phase flow rate and column temperature were maintained at 0.3 mL/min and 30 ℃, respectively. The entire HPLC analysis was performed with an electrospray ionization (ESI) detector at a wavelength of 230 nm for identification compounds; the temperature was 18 °C, and the total flow rate 0.25 mL min^−1^. The injection volume was 10 μL. Additionally, liquid chromatography was combined with a mass spectrometric ion trap for compound identification.

### 4.5. Mass Spectrometry

MS analysis was performed on an ion trap amaZon SL (Bruker Daltoniks, Bremen, Germany) equipped with an ESI source in negative ion mode. MS analysis was carried out in ESI mode using negative and positive polarity for all samples with data-independent MSE acquisition. The optimized parameters were obtained as follows: ionization source temperature: 70 °C, gas flow: 4 L/min, nebulizer gas (atomizer): 7.3 psi, capillary voltage: 4500 V, end plate bend voltage: 1500 V, fragmentary: 280 V, collision energy: 60 eV. An ion trap was used in the scan range *m*/*z* 100−1.700 for MS and MS/MS. The chemical constituents were identified by comparing their retention index, mass spectra, and mass spectrometric fragmentation with a home-library database built by the Group of Biotechnology, Bioengineering and Food Systems at the Far Eastern Federal University (Russia). The capture rate was one spectrum/s for MS and two spectrum/s for MS/MS. Data collection was controlled using Windows software for Bruker Daltoniks. All experiments were replicated in triplicate. A four-stage ion separation mode (MS/MS mode) was implemented.

## 5. Conclusions

The aerial parts of the *D. fragrans* plant contain many polyphenolic constituents and constituents of other chemical groups with valuable bioactivity. Tandem mass spectrometry was applied to detect target analytes. Supercritical CO_2_ extraction of *D. fragrans* was successfully carried out by a team of authors; certain extraction conditions were selected. The extracts obtained showed both a high content of polyphenolic compounds and a high content of the naphthoquinones, diterpenes polysaccharides, triterpenoids, sesquiterpenes, etc. One hundred and forty-one different bioactive compounds were tentatively identified in *D. fragrans* extracts. For the first time, 51 chemical constituents from extracts of aerial parts of *D. fragrans* were tentatively identified. These include flavones, flavonols, anthocyanins, stilbenes pinosylvin and resveratrol, coumarins umbelliferone, fraxetin, 4/6,8-Dihydro-5,7-dihydroxy-2-*oxo*-2H-1-benzopyran-3-acetic acid, umbelliferone hexoside, tomenin, etc. The other newly identified compounds of *D. fragrans* belonged to classes such as naphthoquinones, diterpenoids, polysaccharides, triterpenoids, sesquiterpenes, etc.

The newly obtained mass spectrometric data on the composition of bioactive substances of both polyphenolic and other chemical groups in the aerial parts of *D. fragrans* can be widely used in further research both in the pharmaceutical industry in the development of new medicinal substances and in the cosmetology industry in the development of a wide range of various drugs.

## Figures and Tables

**Table 1:** The yield of bioactive compounds (aerial parts of *D. fragrans*) via CO_2_ extraction.

Pressure	50 Bar	100 Bar	150 Bar	200 Bar	250 Bar	300 Bar
31 °C	0.00000038	0.0000012	0.0000019	0.000002	0.0000023	0.0000026
40 °C	0.0000005	0.0000019	0.0000021	0.0000023	0.0000027	0.0000029
45 °C	0.0000007	0.0000027	0.0000043	0.0000042	0.000004	0.0000035
50 °C	0.0000025	0.0000035	0.000004	0.0000051	0.0000058	0.0000045
55 °C	0.0000026	0.0000037	0.0000038	0.0000062	0.0000059	0.000005
60 °C	0.0000025	0.0000031	0.000004	0.0000045	0.000004	0.0000047

**Table 2:** List of common and specific compounds detected in EtOH, MeOH, and CO_2_ extracts of the aerial parts of *D. fragrans.*

Type of Extraction	Total	Compounds
CO_2_ EtOH MeOH	26	Chrysoeriol *C*-hexoside; Albaspidin PB; Quercetin-*O*-rhamnosyl-hexoside; Chrysophanol; Delphinidin 3-*O*-hexoside; Ellagic acid; Delphinidin 3-*O*-glucoside; Cryptochlorogenic acid; Vitexin; Dihydroxyflavone; Chrysoeriol 6-*C*-glucoside; Chrysoeriol 8-*C*-glucoside; Flavaspidic acid BB; 1,3-Dimethyluric acid; Genistein 6-*C*-glucoside; Delphinidin 3-*O*-β-galactoside; Sinapic acid; Genistein 8-*C*-glucoside; Saroaspidin A; Isovitexin; Linolenic acid; Ethyl protocatechuate; 1,8-Dihydroxy-anthraquinone; Ketoprofen; Chlorogenic acid; Fraxin
EtOH MeOH	24	Kaempferol; Umbelliferone; (R)-eriodictyol-6-*C*-β-*D*-glucopyranoside; Hydroxyferulic acid; Albaspidin PP; Ferulic acid; Luteolin; Hyperoside; Apigenin; Delphinidin 3-*O*-rutinoside; Aspidin AB; Rutin; Eriodictyol-*O*-hexoside; Tomenin; Quercetin 3-*O*-hexoside; Quercetin 3-*O*-glucoside; Trimethoxy flavone; Disflavaspidic acid PB; Loliolide; Delphinidin 3-*O*-(6-*O*-p-coumaroyl) glucoside; 1-*O*-Caffeoylquininc acid methyl ether; Methoxyeugenol; Antheraxanthin; (S)-eriodictyol-6-*C*-β-D-glucopyranoside
CO_2_ EtOH	20	Docosahexaenoic acid; Trihydroxy(*iso*)flavone; Caffeic acid; Quercetin; Delphinidin; 4,8-Trihydroxyanthraquinone; 6-methyl-aloe-emodin; Luteolin 6-*C*-glucoside; Caffeoyl shikimic acid; Herbacetin; Vebonol; Glucaric acid; Luteolin *C*-hexoside; Neochlorogenic acid; (*Epi*)-afzelechin derivative; 2,5-Trihydroxyanthraquinone; Dihydrotanshinone I; Galactaric acid; Luteolin 8-*C*-Glucoside; Astragalin
CO_2_ MeOH	2	Eriodictyol-7-*O*-glucuronide; Trehalose

**Table 3:** Jaccard index for three extracts of *D. fragrans* (ethanol extract, methanol extract, and supercritical CO_2_ extract).

	EtOH Extract (107)	MeOH Extract (64)	CO_2_ Extract (70)
EtOH extract (107)	--	50	46
		0.4132	0.3511
MeOH extract (64)	50	--	28
	0.4132		0.2642
CO_2_ extract (70)	46	28	--
	0.3511	0.2642	

## Data Availability

Data is contained within the article.

## Appendix A

**Table A1:** Chemical compounds identified from the extracts of aerial parts of *D. fragrans* (EtOH extract, MeOH extract, and supercritical CO_2_ extract) in positive and negative ionization modes via ion trap-MS/MS.

	Class of Compounds	Identification	Formula	Calculated Mass	Observed Mass [M−H]^−^	Observed Mass [M+H]^+^	MS/MS Stage 1 Fragmentation	MS/MS Stage 2 Fragmentation	MS/MS Stage 3 Fragmentation	References
		POLYPHENOLS								
1	Flavone	Dihydroxyflavone *	C_15_H_10_O_4_	254.237		255	237; 185; 113	167	125	Chinese herbal formula Jian-Pi-Yi-Shen pill [39]; *Ribes pauciflorum* [34]
2	Flavone	Formononetin [Biochanin B; Formononetol] *	C_16_H_12_O_4_	268.264		269	241; 223	223		*D. jacutense* [47]; *M. varia* [36]; *M. amurensis* [43], *R. fragrans* [42]; Chinese herbal formula Jian-Pi-Yi-Shen pill [39]
3	Flavone	Apigenin [5,7-Dixydroxy-2-(4-Hydroxyphenyl)-4H-Chromen-4-One]	C_15_H_10_O_5_	270.236		271	225	179		*R. meyeri* [29]; *L. japonica* [26]; Andean blueberry [32]
4	Flavone	Trihydroxy(iso)flavone	C_15_H_10_O_5_	270.236		271	196	168	113	Propolis [48]
5	Flavone	Luteolin *	C_15_H_10_O_6_	286.236		287	187; 277			*Inula gaveolens* [49]; *A. absinthium* [50]
6	Flavone	Herbacetin [3,5,7,8-Tetrahydroxy-2-(4-hydro-xyphenyl)-4H-chromen-4-one] *	C_15_H_10_O_7_	302.235		303	275; 203	245; 175	233; 175	*L. caerulea* [51]
7	Flavone	Trimethoxy flavone	C_18_H_16_O_5_	312.316		313	267	239	197; 113	*A. cordifolia* [52]
8	Flavone	Pentahydroxy dimethoxyflavone	C_17_H_14_O_9_	362.287		363	344; 300; 256	238; 146		*G. linguiforme* [52]
9	Flavone	Vitexin [Apigenin 8-C-Glucoside]	C_21_H_20_O_10_	432.377		433	415; 367; 313	337; 283	309	*T. aestivum* [23]; *D. ramosa* [24]
10	Flavone	Isovitexin [Saponaretin; Homovitexin]	C_21_H_20_O_10_	432.377		433	415; 367; 313	337; 283	309	*Passiflora incarnata* [27]; Chilean currants [30]
11	Flavone	Genistein 8-C-glucoside *	C_21_H_20_O_10_	432.377		433	415	337	309	Mexican lupine species [28]
12	Flavone	Genistein 6-C-glucoside *	C_21_H_20_O_10_	432.377		433	415	337	309	Mexican lupine species [28]
13	Flavone	Acacetin 8-C-glucoside *	C_22_H_22_O_10_	446.404		447	429; 377	410; 358; 301; 251	377; 340; 251	Mexican lupine species [28]
14	Flavone	Luteolin 7-O-glucoside [Cynaroside; Luteoloside]	C_21_H_20_O_11_	448.376		449	431; 287	213		*T. aestivum* [23]; *L. japonicum* [26]; *Passiflora incarnata* [27]; Mexican lupine species [28]
15	Flavone	Luteolin 8-C-Glucoside [Orientin; Lutexin]	C_21_H_20_O_11_	448.376		449	431	353; 299	325	*T. aestivum* [23]; *Aspalathus linearis* [25]
16	Flavone	Luteolin 6-C-glucoside [Isoorientin; Homoorientin]	C_21_H_20_O_11_	448.376		449	431	353; 299	325	*T. aestivum* [23]; *D. ramosa* [24]; *Aspalathus linearis* [25]
17	Flavone	Luteolin *C*-hexoside *	C_21_H_20_O_11_	448.376		449	431	353; 299	325	*T. aestivum* [23]
18	Flavone	Eriodictyol-O-hexoside	C_21_H_22_O_11_	450.392	449		287	151		Andean blueberry [32]; *F. glaucescens; F. pottsii* [52]
19	Flavone	(S)-eriodictyol-6-*C*-β-*D*-glucopyranoside	C_21_H_22_O_11_	450.392		451	433	414; 363; 299; 233	344; 258; 213	*Aspalathus linearis* [25]
20	Flavone	(R)-eriodictyol-6-*C*-β-*D*-glucopyranoside	C_21_H_22_O_11_	450.392		451	433	414; 363; 299; 233	344; 258; 213	*Aspalathus linearis* [25]
21	Flavone	6,4′-Dimethoxyisoflavone-7-O-glucoside *	C_23_H_24_O_10_	460.430		461	443; 419; 306	425; 373; 186	407; 257	*Astragali radix* [53]
22	Flavone	Chrysoeriol 6-C-glucoside	C_22_H_22_O_11_	462.403		463	445; 403; 375; 329; 237	426; 401; 347	385; 357; 269	*T. aestivum* [54]
23	Flavone	Chrysoeriol 8-C-glucoside [Scoparin]	C_22_H_22_O_11_	462.403		463	445; 403; 375; 329; 237	426; 401; 347	385; 357; 269	Mexican lupine species [28]; *Citrus sinensis* [33]
24	Flavone	Chrysoeriol *C*-hexoside	C_22_H_22_O_11_	462.403		463	445; 403; 375; 329; 237	426; 401; 347	385; 357; 269	*T. aestivum* [54]
25	Flavone	Eriodictyol-7-O-glucuronide *	C_21_H_20_O_12_	464.376		465	289	271; 163	145	*Mentha* [55]
26	Flavone	Luteolin 8-*C*-pentoside-6-*C*-hexoside	C_26_H_28_O_15_	580.491		581	563; 397; 325	251	223	*T. aestivum* [56]
27	Flavone	Luteolin 8-*C*-hexoside-6-*C*-pentoside	C_26_H_28_O_15_	580.491		581	563; 397; 325	251	223	*T. aestivum* [56]
28	Flavonol	Kaempferol	C_15_H_10_O_6_	286.236		287	269; 149	239; 181		*L. japonica* [26]; *R. meyeri* [29]; Andean blueberry [32]
29	Flavonol	Quercetin	C_15_H_10_O_7_	302.235		303	284	240		*R. meyeri* [29]; Propolis [48]
30	Flavonol	Myricetin	C_15_H_10_O_8_	318.235		319	273; 219	191	209	Andean blueberry [32]
31	Flavonol	Kaempferol 3-O-pentoside	C_20_H_18_O_10_	418.350	417		195	151	136	Andean blueberry [32]; *R. dikuscha* [34]
32	Flavonol	Quercetin 3-*O*-pentoside	C_20_H_18_O_11_	434.350	433		387	301; 231	283	*R. meyeri* [29]
33	Flavonol	Quercetin 3-*D*-xyloside [Reynoutrin]	C_20_H_18_O_11_	434.350	433		387	301	284	Cranberry [31]
34	Flavonol	Quercetin-3-O-arabinoside	C_20_H_18_O_11_	434.350	433		387	301; 233	285	Cranberry [31]
35	Flavonol	Astragalin [Kaempferol 3-O-glucoside] *	C_21_H_20_O_11_	448.376	447		429; 225	183	181	*L. japonica* [26]; *R. meyeri* [29]
36	Flavonol	Kaempferol-3-O-hexoside	C_21_H_20_O_11_	448.376	447		327; 285; 151	255		Andean blueberry [32]
37	Flavonol	Quercitrin [Quercetin 3-O-rhamnoside] *	C_21_H_20_O_11_	448.376	447		327	299		Cranberry [31]
38	Flavonol	Quercetin-3-*O*-hexoside	C_21_H_20_O_12_	464.376	463		301	271; 179	151	*A. absinthium* [50]
39	Flavonol	Hyperoside [Quercetin 3-O-galactoside; Hyperin]	C_21_H_20_O_12_	464.376	463		301	271; 179	151	*T. aestivum* [23]; *L. japonica* [26]; Cranberry [31]; Andean blueberry [32]
40	Flavonol	Quercetin 3-*O*-glucoside [Isoquercitrin; Hirsutrin]	C_21_H_20_O_12_	464.376		465	303	229; 165	201; 161	*T. aestivum* [23]; *R. meyeri* [29]; *L. japonica* [26]; Cranberry [31]
41	Flavonol	Myricitrin *	C_21_H_20_O_12_	464.376		465	447	428; 235	383	Chilean currants [30]
42	Flavonol	Rutin [Quercetin 3-*O*-rutinoside]	C_27_H_30_O_16_	610.517		611	303	257; 165	229	*R. meyeri* [29]; *R. magellanicum* [30]; *L. japonica* [26]
43	Flavonol	Quercetin-*O*-rhamnosyl-hexoside	C_27_H_30_O_16_	610.517		611	303; 465	257; 165	229; 201	Papaya [57]
44	Flavan-3-ol	(Epi)-catechin derivative		379		379	261	233	151	PubChem
45	Flavan-3-ol	(Epi)-afzelechin derivative	C_18_H_16_O_10_	392.313		393	275; 245; 215	245; 175	175; 127	*Z. marina* [35]
46	Flavan-3-ol	(Epi)-catechin derivative		424		425	291	261; 191	191	PubChem
47	Gallotannin	Strictinin [galloyl-HHDP-hexose]	C_27_H_22_O_18_	634.453		635	561; 461	433		*Juglans regia* [58]
48	Anthocyanin	Apigenidin	C_15_H_11_O_4_	255.245		256	182	154	113	*T. aestivum* [59]
49	Anthocyanin	Delphinidin	C_15_H_11_O_7_	303.243		303	257; 165	229		*A. cordifolia* [52]
50	Anthocyanin	Cyanidin-3-O-glucoside	C_21_H_21_O_11_+	449.384		449	287	213	185	*R. magellanicum* [30]
51	Anthocyanin	Cyanidin-3-O-hexoside	C_21_H_21_O_11_+	449.384		449	287	213; 165		Andean blueberry [32]
52	Anthocyanin	Cyanidin-3-*O*-β-galactoside	C_21_H_21_O_11_	449.384		449	287	287; 213	185	*T. aestivum* [23,59]
53	Anthocyanin	Delphinidin 3-O-glucoside	C_21_H_21_O_12+_	465.390		465	303	257; 165	229; 201	*R. magellanicum* [30]
54	Anthocyanin	Delphinidin 3-O-hexoside	C_21_H_21_O_12+_	465.390		465	303	257; 165	229; 173	Andean blueberry [32]; *R. dikuscha* [34]
55	Anthocyanin	Delphinidin 3-O-β-galactoside	C_21_H_21_O_12+_	465.390		465	303	257; 165	229; 173	*R. dikuscha* [34]
56	Anthocyanin	Cyanidin 3-(6”-malonylglucoside) *	C_24_H_23_O_14_	535.431		535	287	241; 165	213	*Medicago varia* [36]; *T. aestivum* [37]
57	Anthocyanin	Cyanidin malonyl hexoside *	C_24_H_23_O_14_	535.431		535	287	241; 165	213	*T. aestivum* [23]; *R. magellanicum* [30]
58	Anthocyanin	Delphinidin-3-O-(6”-O-malonyl)-β-D-glucoside *	C_24_H_23_O_15_	551.430		551	303	257; 165	229	*T. aestivum* [37]
59	Anthocyanin	Delphinidin 3-O-rutinoside [Tulipanin]	C_27_H_31_O_16_	611.525		611	303; 465	257; 165		*L. caerulea* [51]; *R. aureum* [34]
60	Anthocyanin	Delphinidin 3-*O*-(6-*O*-p-coumaroyl) glucoside *	C_30_H_27_O_14_	611.527		611	303; 465	257; 165		*R. pauciflorum* [34]
61	Hydroxycinnamic acid	Caffeic acid [(2E)-3-(3,4-Dihydroxyphenyl)acrylic acid]	C_9_H_8_O_4_	180.157		181	135	119		*R. meyeri* [29]; *L. japonica* [26]
62	Hydroxycinnamic acid	3,4-Dihydroxyhydrocinnamic acid [Dihydrocaffeic acid]	C_9_H_10_O_4_	182.173		183	155	127	145	Chilean currants [30]
63		Ethyl protocatechuate [3,4-Dihydroxybenzoic Acid Ethyl Ester]	C_9_H_10_O_4_	182.173		183	155	126		*Medicago varia* [36]
64	Trans-cinnamic acid	Ferulic acid	C_10_H_10_O_4_	194.184	193		176	132		*L. japonica* [26]; Andean blueberry [32]
65	Hydroxycinnamic acid	Hydroxyferulic acid	C_10_H_10_O_5_	210.183		211	183; 113	165; 113		Andean blueberry [32]; *R. davurica* [60]
66	Hydroxycinnamic acid	Sinapic acid [trans-Sinapic acid] *	C_11_H_12_O_5_	224.21		225	197	152	151	Andean blueberry [32]
67	Hydroxybenzoic acid	Ellagic acid [Benzoaric acid; Elagostasine]	C_14_H_6_O_8_	302.192	301		257; 165	229	201; 173	*R. meyeri* [29]
68	Hydroxycinnamic acid;	Chlorogenic acid [3-O-Caffeoylquinic acid]	C_16_H_18_O_9_	354.308	353		163	145	117	*Artemisia annua* [50]; *R. magellanicum* [30]
69	Hydroxycinnamic acid	Cryptochlorogenic acid [4-O-Caffeoylquinic acid; Quinic acid 4-O-Caffeate]	C_16_H_18_O_9_	354.308	353		163	145	117	*Juglans regia* [58]; *Artemisia annua* [50]; *R. magellanicum* [30];
70		Neochlorogenic acid [5-O-Caffeoylquinic acid]	C_16_H_18_O_9_	354.308		355	163	145	117	*Artemisia annua* [50]; *R. magellanicum* [30]; *L. japonica* [26]
71		Caffeic acid derivative	C_16_H_18_O_9_Na	377.298	377		341	179		*Embelia* [61]
72	Phenolic acid	Ellagic acid pentoside [Ellagic acid 4-O-xylopyranoside]	C_19_H_14_O_12_	434.307	433		387	301; 271	283; 257; 231	*R. pauciflorum; R. aureum* [34]
73	Phenolic acid	Dicaffeoylferuoylquinic acid *		692.345		693	352; 261	261; 123	149	PubChem
74	Phenylpropanoid	1-O-Caffeoylquininc acid methyl ether	C_17_H_20_O_9_	368.335		369	207	192	153	Pear [62]
75	Dihydrochalcone	Phloretin [Dihydronaringenin; Phloretol] *	C_15_H_14_O_5_	274.268		275	257	230	229	*G. linguiforme* [51,52]
76	Stilbene	Pinosylvin [ 3,5-Stilbenediol] *	C_14_H_12_O_2_	212.243		213	167; 139	139		*L. caerulea* [51]; *R. triste* [34]
77	Stilbene	Resveratrol [trans-Resveratrol; 3,4′,5-Trihydroxystilbene] *	C_14_H_12_O_3_	228.243		229	211	183; 127	138	*A. cordifolia; F. glaucescens; F. herrerae* [52]; *R. pauciflorum* [34]
78	Hydroxycoumarin	Umbelliferone [Skimmetin; Hydragin] *	C_9_H_6_O_3_	162.142		163	145	117		*Z. marina* [35]; *L. caerulea* [51]
79	Coumarin	Fraxetin *	C_10_H_8_O_5_	208.167		209	191	117		*Embelia* [61]
80	Coumarin	3,4/6,8-Dihydro-5,7-dihydroxy-2-*oxo*-2H-1-benzopyran-3-acetic acid *	C_11_H_10_O_6_	238.193		239	221	203	185	PubChem
81	Coumarin	Umbelliferone hexoside *	C_15_H_16_O_8_	324.282		325	289; 127	271; 127	253; 146	*F. glaucescens* [52]; *R. triste* [34]
82	Coumarin glucoside	Tomenin *	C_17_H_20_O_10_	384.334		385	223	177	149	Pubchem
83	Coumarin	Fraxin (Fraxetin-8-O-glucoside) *	C_16_H_18_O_10_	370.308		371	209	163; 111	119	*R. rugosa* [60]
85	Lignan	Dimethyl-secoisolariciresinol *	C_22_H_30_O_6_	390.470		391	373; 211	345; 239	299; 247	Lignans [63]
86	Lignan	Podophyllotoxin [Podofilox; Condylox; Condyline] *	C_22_H_22_O_8_	414.405		415	396; 344; 284; 209	378; 326	350	Lignans [63]
	OTHERS									
87		2,3-Dihydro-3,5-dihydroxy-6-methyl-4(H)-pyran-4-one [DDMP]	C_6_H_8_O_4_	144.125		145	127			*Radix polygoni multiflori* [64]
88		Methoxyeugenol	C_11_H_14_O_3_	194.227		195	163	145	117	Ocimum [65]
89		1,3-Dimethyluric acid [Oxytheophylline; 1,3-Dimethylurate]	C_7_H_8_N_4_O_3_	196.163		197	169	113; 151		Coffee [66]
90	Benzofuran	Loliolide	C_11_H_16_O_3_	196.242		197	179	161	133	*D. ramosa* [24]; Jatropha [67]; *A. martjanovii* [68]
91	Naphthoquinone	5,8-Dihydroxy-6-methyl-2,3-dihydro-1,4-naphthalenedione/8-Hydroxy-2-methoxy-2,3-dihydro-1,4-naphthalenedione *	C_11_H_10_O_4_	206.194		207	189	161	133	*J. mandshurica* [69]
92		Galactaric acid [Mucic acid;] *	C_6_H_10_O_8_	210.138		211	193; 165	165		Soybean [70]; *Stevia rebaudiana* [71]
93	Polysaccharides	Glucaric acid [*D*-Glucaric acid; Saccharic acid; *D*-Glutarate] *	C_6_H_10_O_8_	210.138		211	193	165	147	Soybean [70]; Cherimoya, Papaya [57]
94	Aminoalkylindole	5-Methoxydimethyltryptamine *	C_13_H_18_N_2_O	218.294		219	201; 161	159		*Camellia kucha* [72]
95	Sesquiterpenoid	Caryophyllene oxide [Caryophyllene-alpha-oxide] *	C_15_H_24_O	220.350		221	203; 163	135		*R. davurica* [60]
96	Naphthoquinone	1,8-Dihydroxy-anthraquinone [Chrysazin] *	C_14_H_8_O_4_	240.210		241	213	195		*J. mandshurica* [69]
97	Peptide	5-*oxo*-L-propyl-L-isoleucine	C_11_H_18_N_2_O_4_	242.271		243	197	169	113	*Solanum tuberosum* [73]
98	Propionic acid	Ketoprofen [Orudis; 2-(3-Benzoylphenyl) propionic acid; Profenid] *	C_16_H_14_O_3_	254.280	253		209; 191; 165; 141	194; 167; 124	165; 125	*Ginkgo biloba* [74]
99	Naphthoquinone	Chrysophanol [Chrysophanolic acid] *	C_15_H_10_O_4_	254.237		255	237; 112	112		Chinese herbal formula Jian-Pi-Yi-Shen pill [39]
100	Naphthoquinone	1,4,8-Trihydroxyanthraquinone *	C_14_H_8_O_5_	256.210		257	183; 113	155	113	*J. mandshurica* [69]
101	Naphthoquinone	1,2,5-Trihydroxyanthraquinone *	C_14_H_8_O_5_	256.210		257	183; 113	155	113	*J. sigillata* [75]
102	Omega-3-fatty acid	Stearidonic acid [6,9,12,15-Octadecatetraenoic acid]	C_18_H_28_O_2_	276.413		277	257	229	187	*G. linguiforme* [52]; *R. triste* [34]
103		Dihydrotanshinone I *	C_18_H_20_O_3_	278.302		279	261; 123	177	135	*Salviae Miltiorrhizae* [40]
104	Omega-3-fatty acid	Linolenic acid (Alpha-Linolenic acid; Linolenate)	C_18_H_30_O_2_	278.429		279	261; 219; 177	177; 219	135	*Salviae Miltiorrhizae* [41]; *M. amurensis* [43]
105	Sesquiterpenoid	Artemisinin [Arteannuin; Artemisinine] *	C_15_H_22_O_5_	282.332	281		237	235	191	*A. absinthium* [50]; *A. martjanovii* [68]
106	Omega-9 unsaturated fatty acid	Oleic acid (Cis-9-Octadecenoic acid; Cis-Oleic acid)	C_18_H_34_O_2_	282.461	281		237	235	191	Huolisu Oral Liquid [38]
107	Dihydroxy anthraquinone	6-methyl-aloe-emodin	C_16_H_12_O_5_	284.263		285	211; 141	183; 113	165	Chinese herbal formula Jian-Pi-Yi-Shen pill [39]
108	Isocoumarin	Brevifolincarboxylic acid *	C_13_H_8_O_8_	292.197	291		248	205	204; 146	*P. granatum* [76]; *Carpinus betulus* [77]
109		1-(3,4-Dihydroxyphenyl)-3-(2,4,6-trihydroxyphenyl)-2-propanol	C_15_H_16_O_6_	292.283	291		248	205; 178	148	*Ribes magellanicum* [30]
110	Diterpenoid	Tanshinone IIA * [Tanshinone II; Tanshinone B]	C_19_H_18_O_3_	294.344		295	277	259; 175	199	Huolisu Oral Liquid [38]; *Radix Salviae* [40]
111	Diterpenoid	Cryptotanshinone *	C_19_H_20_O_3_	296.360		297	279	261; 177	177	Huolisu Oral Liquid [38]; *Radix Salviae* [40]
112	Diterpenoid	Isocryptotanshinone II *	C_19_H_20_O_3_	296.360		297	279	261; 177	177	*Salviae Miltiorrhizae* [41]
113	Diterpenoid	Tanshinone IIB [(S)-6-(Hydroxymethyl)-1,6-Dimethyl-6,7,8,9-Tetrahydrophenanthro[1,2-B]Furan-10,11-Dione] *	C_19_H_18_O_4_	310.343	309		237	222	180	*Salviae Miltiorrhizae* [41]; Huolisu Oral Liquid [38]
114	Diterpenoid	7-Dehydroabietic acid	C_20_H_26_O_3_	314.418		315	287	187; 259		*D. ramosa* [24]
115	Long-Chain Polyunsaturated Fatty Acid	Docosahexaenoic acid [Doconexent; Cervonic acid]	C_22_H_32_O_2_	328.488		329	311; 283; 255	283		PubChem
116	Cyclohexenecarboxylic acid	Caffeoyl shikimic acid	C_16_H_16_O_8_	336.293		337	301; 193	255; 227; 173	199; 170	*R. aureum, R. pauciflorum* [34]; *Carpinus betulus* [77]
117	Cyclohexenecarboxylic acid	4-O-caffeoyl shikimic acid	C_16_H_16_O_8_	336.293	335		317	273; 205	189; 121	*Inula viscosa* [77,78]
118	Naphthoquinone	4,8-Dihydroxy-1-naphthyl-beta-*D*-glucopyranoside	C_16_H_18_O_8_	338.309		339	147	119		*J. mandshurica* [68,69]
119	Disaccharide	Trehalose	C_13_H_24_O_13_	388.321	387		341	178		Pubchem
120	Sterol	Fucosterol [Fucostein; Trans-24-Ethylidenecholesterol]	C_29_H_48_O	412.690		413	395; 268; 209	378; 254	350; 208	*F. pottsii* [52]; *L. caerulea* [51]
121	Sterol	Beta-Sitostenone	C_29_H_48_O	412.690		413	209; 149	191; 149	149	*F. herrerae* [52];
122	Steroidal alkaloid	Solasodine	C_27_H_43_NO_2_	413.635		414	396; 209	378; 326	350	*Jatropha* [67]
123	Phlorodlucinol derivative	Disflavaspidic acid PB	C_23_H_28_O_8_	432.463		433	415	337	309	*D. fragrans* [20]
124	Phlorodlucinol derivative	Aspidin AB	C_23_H_28_O_8_	432.463		433	415	337	309	*D. fragrans* [20]
125	Phlorodlucinol derivative	Albaspidin PP	C_23_H_28_O_8_	432.463		433	415	337	309	*D. fragrans* [20]
126	Amorphygenin	Dalbinol [12A-Hydroxyamorphygenin]	C_23_H_22_O_8_	426.416		427	409; 291; 233	339	241; 173	*D. ramosa* [24]
127	Pentacyclic triterpene	Lupeol [Fagarasterol; Clerodol; Lupenol] *	C_30_H_50_O	426.717		427	409; 291; 233	339	241; 173	*J. mandshurica* [69]
128	Triterpenoid	Uvaol	C_30_H_50_O_2_	442.716		443	425; 219	206; 151		*F. herrerae* [52]
129	Phlorodlucinol derivative	Albaspidin PB	C_24_H_30_O_8_	446.490	445		427; 401; 347; 247	385; 341; 297; 247	368; 343; 398; 283	*D. fragrans* [20]
130	Phlorodlucinol derivative	Flavaspidic acid BB	C_24_H_30_O_8_	446.490	445		401; 223	179; 153	150	*D. fragrans* [20]
131	Phlorodlucinol dimer	Saroaspidin A	C_24_H_30_O_8_	446.490	445		427; 401; 347; 247	385; 341; 297; 247	368; 343; 398; 283	*D. fragrans* [20]
132	Anabolic steroid	Vebonol *	C_30_H_44_O_3_	452.668		453	435; 210	226; 336	210	*H. polyrhizus* [79]
133	Triterpenoid	Betulonic acid *	C_30_H_46_O_3_	454.684		455	438; 237	420; 321; 248; 159	375	*R. rugosa* [60]
134	Triterpenic acid	Ursolic acid *	C_30_H_48_O_3_	456.700		457	439; 223	421; 209	379; 268	*J. mandshurica* [69]; Ocimum [65]; Pear [62]
135	Triterpenoid	1-Hydroxy-3-oxours-12-en-28-oic acid	C_30_H_46_O_4_	470.683		471	453; 237	435; 383	417; 365	Pear [62]
136	Cyclohexenecarboxylic acid	Caffeoylquinate shikimate derivative		510.618		511	493; 317	475; 282	457; 405	*Phoenix dactylifera* [80]
137	Indole sesquiterpene alkaloid	Sespendole *	C_33_H_45_NO_4_	519.714		520	184	125		*H. polyrhizus* [79]
138		Eucaglobulin B *	C_25_H_34_O_12_	526.530		527	509; 351; 182	464; 291	418; 139	*Eucalyptus genus* [81]
139	Carotenoid	Antheraxanthin [All-Trans-Antheraxanthin]	C_40_H_56_O_3_	584.870		585	566; 377; 237	548; 475; 381	485; 389; 259	Carotenoids [82]
140		(all-E)-violaxanthin butyrate		670.123		671	653; 431	575; 353	353	Carotenoids [82]
141	Steroidal alkaloid	Alpha-solanine	C_45_H_73_NO_15_	868.958		868	706; 560; 398	327; 157		*S. tuberosum* [83]

* Chemical compounds identified for the first time in *D. fragrans.*
